# Rotavirus Vaccination Coverage among Children Aged 2-59 Months: A Report from Guangzhou, China

**DOI:** 10.1371/journal.pone.0068169

**Published:** 2013-06-28

**Authors:** Qing He, Ming Wang, Jianxiong Xu, Chunhuan Zhang, Hui Wang, Wei Zhu, Chuanxi Fu

**Affiliations:** 1 Guangzhou Center for Disease Control and Prevention, Guangzhou, Guangdong, China; 2 Southern Medical University, Guangzhou, Guangdong, China; Glaxo Smith Kline, Denmark

## Abstract

**Objective:**

We aimed to estimate the Lanzhou lamb rotavirus (LLR) vaccination coverage (VC) and timeliness among children aged 2 to 59 months in Guangzhou, China.

**Methods:**

An electronic system-based VC survey was conducted using stratified cluster random sampling.

**Results:**

We reported an overall Lanzhou lamb rotavirus vaccine coverage of 25.3% among children aged 2-59 months (2-8 months, 2.6%) in Guangzhou, China.

**Conclusion:**

Great efforts should be taken to increase LLR VC in eligible children in Guangzhou, China.

## Introduction

Rotavirus infection is a common disease in children, which can lead to severe dehydration, hospitalization, and even death in babies and young children [1]. Globally, rotavirus infection is responsible for 453,000 deaths in children under 5 years old, accounting for 37% of diarrheal deaths and 5% of all deaths in children under 5 [2]. In China, approximately 47.8% of children with diarrhea are hospitalized because of rotavirus infection [3]. Approximately 134,000 children younger than 5 years died from rotavirus and 70% of deaths occurred in rural areas [4]. The health consequences of rotavirus were estimated to cost nearly 61.4 million USD per year [5].

The Lanzhou lamb rotavirus (LLR) vaccine is the only vaccine approved for rotavirus infection prevention in China. From 2001 to 2011, children received 838,780 doses of LLR vaccine in Guangzhou [6]. However, there is a paucity of publicly available data regarding this vaccine. Therefore, we conducted an electronic system-based vaccination coverage (VC) survey in Guangzhou to estimate the LLR VC and timeliness among children aged 2 to 59 months to optimize the LLR immunization schedule.

## Methods

The ethics committee of Guangzhou Center for Disease Control and Prevention (Guangzhou CDC) reviewed and approved this protocol. Health children’s vaccination data used in our analysis is based on a large electronic system in Guangzhou CDC instead of records from hospitals and the ethics committee agreed that the oral or written consent are not needed in this analysis.

The cross-sectional survey was performed in January 2013 using the Guangzhou Children’s Expanded Programmed Immunization (EPI) Administrative Computerized System, which was designed to manage children’s vaccination information, as described previously [7]. A stratified cluster random sample of 8400 children born from January 2008 to October 2012 was derived from the system. More precisely, Guangzhou was divided into three areas by economic status (urban, rural and rural-urban continuum). In each area, prefectures were randomly selected with probability proportional to 2011 population size. The child population in each prefecture was considered a study population. A cluster of 200 children was derived from the population by age group (2-5, 6-8, 9-11, 12-17, 18-23, 24-35 and 36-59 months) and gender (male and female) randomly. Children’s LLR vaccination information was collected from the Children’s EPI Administrative Computerized System.

The percentage of children who were vaccinated was reported along with 95% confidence intervals (CIs). We performed a χ^2^ test or Fisher’s exact test to compare the VC of different groups. Multivariate logistic regression analyses were used to examine the association between covariates (age group, gender and living area) and the receipt of 1 or 2 dose of LLR. SPSS statistical software (version 13.0, SPSS, Inc., Chicago, IL) was used for data validation and statistical analysis. We set the level of significance as two-sided *P*<0.05.

## Results

We determined LLR VC for 8400 children aged 2 to 59 months, of which the median age was 16.0 months. Overall, 2122 (25.3%) of children had received at least 1 dose of LLR vaccine. Among children who received LLR vaccine, 1904 (89.7%) received 1 dose of LLR, and only 208 (9.8%) and 10 (0.5%) received 2 or 3 doses, respectively. The median vaccination age of the first, second and third dose was 12.0, 26.0, and 38.0 months, respectively.

Of 2122 children who had received LLR vaccination, 235 (11.1%) were administered in 2-5 months old, 237 (11.2%) in 6-8 months old, 522 (24.6%) in 9-11 months old, 867 (40.9%) in 12-17 months old, 134 (6.3%) in 18-23 months old, and 123 (5.8%) in 24-59 months old. It is worthwhile to note that 4 (0.2%) children had received LLR vaccination at the age of 0-1 month.

LLR VC was very low among the youngest subgroup of children aged 2-5 months, at 1.2% (95% CI, 0.6-1.8%), and 3.9% (95%CI, 2.8-5.0%) among children aged 6-8 months. LLR VC levels varied significantly by age group, area and year. The children aged 36-59 months, children in rural areas, and children born in 2009 had higher VC levels (Table 1).

**Table 1:** LLR vaccination coverage among children ages 2-59 months in Guangzhou^*^.

	n	Denominator	% (95%CI)	P Value
**Age (in months)**				<0.001
2-5	14	1200	1.2(0.6,1.8)	
6-8	47	1200	3.9(2.8,5.0)	
9-11	98	1200	8.2(6.6,9.7)	
12-17	321	1200	26.8(24.2,29.3)	
18-23	480	1200	40.0(37.2,42.8)	
24-35	562	1200	46.8(44.0,49.7)	
36-59	600	1200	50.0(47.2,52.8)	
**Gender**				0.76
Male	1055	4200	25.1(23.8,26.4)	
Female	1067	4200	25.4(24.1,26.7)	
**Area**				<0.001
Urban	690	2800	24.6(23.0,26.2)	
Rural	809	2800	28.9(27.2,30.6)	
Rural-urban continuum	623	2800	22.3(20.7,23.8)	
**Year**				<0.001
2008	28	600	4.7(3.0,6.4)	
2009	255	600	42.5(39.0,46.5)	
2010	301	1200	25.1(22.6,27.5)	
2011	546	2400	22.3(21.1,24.4)	
2012	992	3600	27.6(26.1,29.0)	
Total	2122	8400	25.3(24.3,26.2)	

* Only one dose of vaccination was analyzed.

After adjusting for covariates, multivariate logistic regression models showed that children in 2-8 months were less likely than children in 9-11 and 12-17 months to receive 1 or 2 doses of LLR vaccine. Children in rural area were more likely to receive LLR vaccines. No significant difference was observed between genders (Table 2).

**Table 2:** Factors associated with LLR vaccine uptake among children ages 2-59 months.

	Received 1 Dose^*^, OR (95% CI)	Received 2 Doses, OR (95% CI)
**Vaccination age (month)**		
2-8	Ref	Ref
9-11	2.34 (2.03, 2.70)	3.34 (2.42, 4.62)
12-17	4.88 (4.25, 5.59)	15.14 (11.45, 20.02)
18-23	0.93 (0.75, 1.14)^#^	26.05 (19.78, 34.31)
24-35	0.88 (0.71, 1.10)^#^	35.22 (26.77, 46.34)
36-59	0.08 (0.04, 0.16)	31.93 (24.20, 42.12)
**Gender**		
Male	Ref	Ref
Female	0.96(0.87,1.07)^#^	1.02 (0.91, 1.14)^>#^
**Area**		
Urban	Ref	Ref
Rural	1.22 (1.08, 1.39)	1.30 (1.14, 1.49)
Rural-urban continuum	0.88 (0.78, 1.01)^#^	0.85 (0.74, 0.97)

* Excluding 4 children who received LLR vccine at the age of 0-1 month.

# P>0.05

## Discussion

We describe an LLR VC of 25.3% among children age 2-59 months in Guangzhou, China in 2013 and find that 89.7% of these children received only 1 dose of LLR vaccine. This reveals (i) an extremely low LLR VC in the younger children (2-5 months, 1.2%; 6-8 months, 3.9%), (ii) late vaccination in eligible children (2-5 months, 11.1% and 6-8 months, 11.2%), and (iii) unnecessary vaccination in some children (0-1 month, 0.2% and 24-59 months, 5.8%).

In our previous case-control study conducted in Guangzhou, the percentage of vaccinated controls was 22.9% among children aged 2-59 months in 2007 and 16.8% among children aged 2-35 months in 2012 [6,8], which are slightly lower than current findings (25.3% among 2-59 months, 21.1% among 2-35 months). Another electronic system-based survey reported a VC of 39.7% among children aged 14-61 months in Beijing in 2012 [9], which is similar to the findings of this study (12-59 months, 40.9%). The national, state and local area rotavirus VC in United States was 59.2% in 2010 and 67.3% in 2011 [10], which is much higher than ours (3.9% among 6-8 months, rotavirus vaccination is only administered to children up to 8 months old in United States).

Although LLR vaccination activity in China has progressed over the last 10 years, the current LLR VC is still very low in the target children (2-8 months). This may be caused by several factors. A major determinant is the unclear schedule recommendation of LLR vaccine, which recommends that children receive vaccine at 2-35 months. In addition, most children (94.5%) have rotavirus diarrhea during the first 2 years of life, 16.9% have rotavirus by 6 months old, and 59.1% by 1 year old [3]. Therefore, it is essential for children to receive vaccination as soon as possible to ensure the induction of protection prior to natural rotavirus infection. A 2-year-old may not need to receive the LLR vaccination.

**Figure 1:**
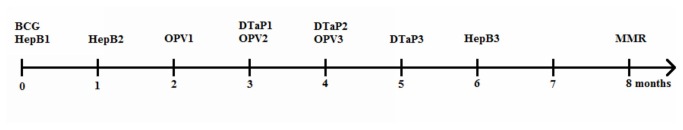
The National Immunization Program schedule for children in China. BCG: bacillus Calmette–Guérin HepB: hepatitis B vaccine OPV: oral poliomyelitis vaccine. DTaP: diphtheria, tetanus, and pertussis MMR: measles, mumps, and rubella vaccine.

Another crucial factor contributing to the low levels of LLR VC is the full immunization schedule from birth through 8 months (Figure 1). Unfortunately, no data are available on LLR vaccine’s combined immunization with EPI vaccines in China. In the United States, Latin America, Europe, South Africa and Singapore, a series of studies showed that rotavirus vaccine was efficacious and well-tolerated when administered with routine vaccines and did not impair the immunogenicity of any co-administered vaccines [11–15]. The studies also indicated that the immune seroconversion response of three doses of rotavirus vaccine was not superior to two doses [11–15]. Hence, we suggest that studies on LLR immunization combined with routine vaccines be performed in China. If possible, children should receive two dose of LLR vaccine, co-administered with routine EPI vaccines during 2-8 months of age.

The third possible reason may be the lack of awareness regarding rotavirus vaccination among parents in China. Some parents have never heard of the LLR vaccine and do not realize the seriousness of rotavirus gastroenteritis in children. The high cost of LLR may also affect the uptake of LLR. The LLR vaccine has not been introduced to the National Immunization Program in China, and the vaccine is priced at sixty-three point four eight USD for two doses, which is almost a tenth of a worker’s average monthly salary in China (International Labor Organization, 2012). In our results, only 218 (10.3%) children received 2 or 3 doses of LLR vaccine. Therefore, parents should be educated regarding rotavirus and the vaccination, and a more effective, safe and less expensive rotavirus vaccine should be developed in China.

In Shenzhen, China, Li et al. conducted a LLR field trial in children age 2-35 months and followed up for 1-11 months [16]. Their results showed that vaccinated children had a significantly lower incidence of diarrhea than unvaccinated children (2.1% vs. 8.4%). The diarrhea cases in vaccinated children were less severe than unvaccinated children (mild, moderate, severe outcomes accounted for 100%, 0%, 0% vs. 69.2%, 23.1%, 7.7%). A series of case control studies in Guangzhou conducted by Fu et al. revealed that vaccine effectiveness is 43.8-77.0% in preventing rotavirus gastroenteritis [6,8,17]. These post-licensure studies of LLR indicate that earlier and full vaccination can prevent children from rotavirus infection or relieve diarrhea outcomes.

This observational study has some limitations. First, we were unable to obtain the VC of floating children who were not registered in the system. These children might be less likely to receive LLR, which could overestimate the VC in our results. Second, we used area economic development instead of family economic status during our sampling process, meaning that economic development may vary by family in an area.

Due to high VC in US, a marked reduction in diarrhea and rotavirus hospitalizations among children was observed after the introduction of rotavirus vaccine in 2006 [18]. Because of herd immunity due to rotavirus vaccination, in addition to the direct effects of vaccination, indirect effects were also noted in children who did not receive the vaccine [18]. Consequently, eligible children should be encouraged to receive LLR vaccine in time to prevent rotavirus gastroenteritis.

In order to increase LLR VC to a higher level, great efforts should be taken to encourage eligible children to receive LLR vaccine as early as possible, to enhance the parents’ awareness of rotavirus gastroenteritis and LLR vaccination. Studies on LLR vaccine coadministered with routine vaccines should be conducted. Moreover, a more effective, safe and less expensive rotavirus vaccine should be developed in China.
